# Supplementary material for: CBEA: Competitive balances for taxonomic enrichment analysis
Source: PLoS Comput Biol. 2022 May 18;18(5):e1010091. doi: 10.1371/journal.pcbi.1010091 (PMC9154102; doi:10.1371/journal.pcbi.1010091)
Supplement: Attachment — Submitted filename: CBEA_reviewer_response.pdf [file pcbi.1010091.s009.pdf]

CBEA: Competitive balances for taxonomic enrichment analysis

PCOMPBIOL-D-21-01648

Response to Reviewers

Feb 15th, 2022

We greatly appreciate both reviewers for thorough and insightful reviews of our manuscript. In response, we have made extensive changes to the manuscript that have greatly improved it. Here, we summarize the major changes, with point-by-point responses to each reviewer comment in the following sections:

1) We adjusted the title of our manuscript to better reflect the proposal and its relationship with existing methods. The title of the manuscript will now be "CBEA: Competitive balances for taxonomic enrichment analysis". This is in response to comment #1B from Reviewer 1. For the remainder of this response document, we will be referring to our approach as CBEA instead of cILR.
2) We restructured the introduction section of the manuscript to condense unnecessary details while also adding detail related to issues raised by reviewers.
    a. We added more discussion and clarification on the compositional nature of microbiome relative abundance data as motivation to our approach. This was also done in response to requests for clarification from Reviewer 1 in comment #1A and #1D.
    b. We provided a more comprehensive overview of gene set testing methods, distinguishing between the different sub-types. This will address some confusion about why ssGSEA and GSVA were chosen as comparison methods from Reviewer 1 in comment #3A.
    c. We expanded on the existing section regarding why our approach is better motivated to solve current issues with set-based testing of microbiome relative abundance data, particularly expanding on the issue of how taxon-specific biases impact sum-based aggregation methods as was requested by Reviewer 1 in comment #1A.
    d. We provided more signposting for the organization of the manuscript as suggested by Reviewer 1 in comment #4B.
3) We added sections in the material and methods section to provide more clarity and precision in the discussion of our statistical motivation.
    a. We provided additional detail on the relationship between our method and the isometric log-ratio transformation (ILR), clarifying that our approach is not a complete transformation (hence the change in the name of our approach) in response to comments from Reviewer 1. We provided comparisons between our approach and alternate methods based on the ILR such as PhILR [1], and phylofactor [2]. This is in response to comment #1B from Reviewer 1.
    b. We provided additional details on the issue of variance inflation due to inter taxa correlation. We added an explainer on the motivation of the issue (as raised by Reviewer 1 in comment #1G), discussed how correlation might inflate the null distribution of our test statistic, and provided additional considerations where a user might not need to adjust for correlation (as raised by Reviewer 2 in comment #2).

4) We reorganized the results section of the manuscript and added further analyses to align our evaluation strategy with existing standards for benchmarking gene set testing methods (as suggested by Reviewer 2). Our evaluation mainly involves analyzing real data sets, so we moved all our simulation analyses into the supplemental section. We still discuss these results where relevant [3].
    a. We agree with Reviewer 1 in comment #4B that differential abundance using set features is equivalent to enrichment analysis at the population level. As such, we have re-labeled "single-sample enrichment testing" to "Inference at the sample level" while the differential abundance section is now titled "Inference at the population level". Both sections are now grouped together under the general enrichment analysis umbrella.
    b. We have re-organized the results section into three evaluation tasks: statistical inference (type I error evaluations and other analyses performed under the null), phenotype relevance (power and sample ranking analyses), and downstream analyses (prediction analyses). As stated in part a), each of the sections on statistical testing will be subdivided into inference at the population level and inference at the sample level.
    c. We have also added additional evaluations based on standards set by the reference [3] suggested in comment #2 from Reviewer 2. For "inference at the sample level", our type I error real data evaluation will be using random set analyses which correspond to the stated null scenario of CBEA This change is in response to Reviewer 1's comment #3B. For "inference at the population level", we added random set analyses in addition to the label permutation analyses already performed in the original manuscript.
    d. We provided more explicit language to describe the run-time analyses conducted in the supplementary section of our manuscript.
5) We have revised the discussion section to include interpretations on results from the newly added analyses.
    a. We provided a discussion on situations where CBEA would have the best performance based on results obtained under different evaluation scenarios. This provides the additional context necessary to evaluate our model while also helping potential users apply our approach to their own data.
    b. We condensed the existing explanation sections to avoid repeating information while also being more precise regarding the performance of our approach (in response to comment #3A from Reviewer 1).
    c. We added new discussion in light of the additional analyses performed as a response to comment #2 from Reviewer 2.
    d. We added more clarification on the limitations of our evaluation and method, including:
        i. Highlighting the difficulty of evaluating power/phenotypic relevance of enrichment methods and clarifying how our evaluation data set can address those goals in response to comment #2 from Reviewer 2 and comment #3B from Reviewer 1.
        ii. Limited ability to address data sparsity in response to comment #2A from Reviewer 1.

We believe these changes in response to reviewer comments have made the manuscript more focused and better aligned with previous benchmarking standards, while additionally providing the needed clarity and precision of language around complex statistical terminology. Our revisions also highlight the main contribution of the manuscript which is fundamentally a single sample set enrichment analysis approach (in the same vein as ssGSEA). In the following section we provide explicit responses to comments given by reviewers. Reviewer comments are italicized while our responses are in unformatted text. Reviewer comments are organized according to the original text to the best of our ability.

Reviewer 1:

<u>*Summary*</u>

In this manuscript Nguyen et al propose cILR for a set-enrichment-like analysis of microbiome sequencing data. This is a scale invariant alternative to the more standard (and problematic) approach of applying GSEA to DESeq2 output or other such approaches. Overall, it's a nice idea and is fairly well executed. The statistical analysis is largely appropriate, and it could be a nice contribution. My largest comments relate to the lack of precision in the authors' writing, the extent of the authors claims, and the authors handling of zeros and count variation.

We greatly appreciate the reviewer's detailed comments and suggestions for improving the manuscript. We have made various changes to the manuscript to clarify the concepts we are advancing and provide more context while interpreting our results to avoid confusion. In the following sections we provide detailed responses to each of the reviewer's concerns.

<u>*Detailed Comments*</u>

1. *Precision of Writing:*

   A. *The authors repeatedly state this data is "compositional" or "strictly compositional". First, what is "strictly compositional" is there non-strictly compositional data? Second, the data is clearly not compositional; this is a cliche that has been amplified in the literature and is incorrect. The data is count data, it has zeros and is integer valued, both of those features are in strict contrast to the standard definition of compositional data (i.e., positive multivariate continuous data that sums to a constant value and typically is an open set excluding zero to avoid issues with log-ratio transforms). This distinction is non-trivial as the direct application of log-ratio transforms to this data is poorly motivated in this case and fraught with problems (See later comments on handling of the data).*
   We would like to thank the reviewer for the comments with respect to the clarity of our claims around the compositional nature of the data. We agree that the terminology used in the manuscript was confusing, and it was not clear how we reached the assumption that microbiome data is compositional. We adjusted the introduction section to provide more context behind this motivation and have avoided confusing terms such as "strictly compositional". Here, we provide a discussion on this issue that was also included in the manuscript (see paragraph 4 of the introduction section). We feel that this is also a response to the reviewer's comment below (#1D) since both comments (#1A, #1D) refer to this issue of compositionality.

   We agree with the reviewer that we cannot get absolute abundances of features measured via sequencing data and that count data alone is not compositional according to Aitchison's definition [4]. However, despite efforts to sequence equimolar amounts of DNA as the reviewer noted, the total number of sequences per sample (i.e., library size) still varies significantly across samples [5,6] and requires applying normalization approaches to ensure that abundances can be compared across samples [7]. The most used normalization approach is to transform counts into proportions using the total library size per sample as the denominator. Even though some researchers suggest

using existing methods in the gene expression literature [8], some of the assumptions that underlie these approaches might not match that of microbiome data. For example, DESeq2's median of ratios method (in the function *estimateSizeFactors*) assumes that the majority of genes do not differ in expression levels across samples. Other studies have also empirically compared different normalization methods, where transformation to proportions is usually the best choice [9]. As such, we mistakenly used the term "strictly compositional" to refer to the fact that microbiome sequencing data, unlike other sequencing data sets, is usually transformed to proportions prior to analysis (as stated above, we have removed this terminology from the manuscript). In the case where researchers transform count data into proportions, the data becomes compositional as a sum constraint has been imposed. Even though there are zeroes in the composition, which does not fit Aitchison's definition, the imposed sum to one constraint still induces spurious negative correlation between the variables, where log-ratio based methods are well motivated as solutions [5]. Our approach is conceptually a log-ratio based method for aggregating compositional variables. This concept is not novel as it has been advanced previously by the original authors of the ILR transformation [10], where it was termed as balances between groups of parts. Our contribution is towards specifying the "groups" that has an interpretation similar to that of the competitive null hypothesis in the gene set testing literature.

B. *What the authors propose is not an ILR transform. Unless I am mistaken, there is no constraint on the matrix A such that the coordinate system is cartesian with an orthonormal basis. In fact, if k does not equal p-1 then it cannot possibly be isomorphic let alone isometric with respect to the Aitchison metric. Unless I am mistaken, the authors should change the name of their method and modify their discussion to be more accurate. I would relate this method is not an ILR transform but it is very similar to phylofactor which takes a similar approach (in phylofactor set membership is dictated by the topology of the tree).*

We agree with the reviewer that the method itself is not an ILR transform (as it did not propose a novel binary partition such as PhILR [1]) and was therefore mislabeled. Our approach still leverages the concept of balances between groups of compositional parts related to the ILR transformation as advanced by the original authors [10]. As such, our approach will be renamed "Competitive compositional balances for taxonomic enrichment analysis" (CBEA). We revamped the first section of "Properties of CBEA" to reflect this distinction and provide more discussion on CBEA's influences. We hope that this new name more clearly reflects the specific advances our method is proposing.

C. *The authors state that the "data is zero-inflated" this is another cliche that I would encourage the authors to remove. Zero-inflation is a particular family of models for these zeros not a objective characteristic of the data. Simply saying there are many zeros would likely suffice in this article. They could argue that the data generating mechanism is well represented by a zero-inflation process, but this has been called into question (see Silverman et al. Naught all zeros in sequence count data are the same.)*

We agree with the reviewer that the term "zero-inflated" should be used in reference to the specific class of models instead of as a catch-all term for a characteristic of the data. Since we are agnostic to the mechanism behind the process of generating zeroes, we have amended the article to use "zero abundant" or "sparse".

D. *The authors state that the data is compositional because the number of reads obtained is constrained by the sequencing instrument. Would an instrument that didn't have this constraint lead to "non-compositional data"? This seems unlikely. For example, standard equimolar pooling protocols explicitly dilute concentrated DNA from each sample to try to equalize sequencing depth. It's not just an issue of the sequencer. Even sampling from an environment (e.g., taking 5 grams of stool from a larger stool sample) loses the notion of absolute abundance.*

We agree with the reviewer that the terminology is confusing and have added additional clarity to the manuscript. We have provided a more comprehensive response on the issue of the compositional nature of microbiome data in the above section (response to comment A), which we believe also addresses the issues raised in this comment.

E. *The GSEA method cited on line 51 is not a random-walk like statistic. I think it may be a Brownian bridge but its constrained to be zero at either end -- not a random walk.*

We thank the reviewer for the clarification. The "random-walk like statistic" phrase has been clarified and amended in the introduction section.

F. *Between lines 73 and 85 the authors do not properly motivate the multiplicative rather than additive amalgamation. They mention the downsides of the "naive summation-based method" but this is unclear. From later in the manuscript I gather that this statement reflects the perturbation invariance of multiplicative amalgamation: given that some have argued that measurement bias can be modeled as a constant compositional perturbation. This needs to be made explicit. There is no inherent downside of summation (i.e., additive amalgamation) - its a modeling choice and it is not "naive".*

We have reworked the introduction section (see paragraph 5) to highlight the differences more clearly between product and sum-based aggregations and provide a robust justification for our approach. We have also removed the characterization of sum-based amalgamations as "naïve" as suggested.

G. *The authors mention "adjusting for correlation" multiple times throughout the manuscript yet the motivation is not properly clarified. The best I can guess is that they are saying that they need to modify the null-hypothesis to account for a trivial case where something looks differential expressed or set enriched when really it's just due to the correlation structure between taxa. That said, I think there are many potential sources of confusion that the authors should clarify. Couldn't set enrichment be reflected in those correlations? Isn't the correlations actually a non-trivial part of what the authors are trying to model? In other words if a set of microbes is highly correlated wouldn't that be a sign that that set is potentially enriched or de-enriched? I don't think I understand this point completely but I think it is likely non-trivial. I would encourage the authors to clarify the role of correlation.*

We thank the reviewer for pointing out that the motivation behind "adjusting for correlation" was not clearly communicated in the manuscript. We have amended the "Statistical properties" part of the manuscript and introduce a separate section to provide more clarity on this concept.

Additionally, we agree with the reviewer that there are situations where highly correlated sets are biologically relevant. As such, we have provided additional discussion with regards to that issue and have left the decision whether to adjust for correlation to the user. This also supports the notion (as also recommended by Reviewer 2) that set-based analysis is usually exploratory rather than confirmatory so an inflated type I error may be acceptable to achieve higher power.

H. *On lines 167-170 the authors state that since the cILR are not orthogonal a correlation can exist between cILR aggregated variables. This is misleading there can be correlation whether or not the cILR's are orthogonal or not, orthogonality and a lack of correlation are separate concepts.*

In the statistical properties section, we have removed this distinction due to the fact that CBEA is not a complete transformation, which will correct for this misconception.

I. *Citation on line 187. I don't see how this paper supports this statement. Egozcue et al. take a almost purely mathematical approach as far as I can tell do not discuss central limit theorems or other things that are implied by the authors statement. If I remember correctly the relevant citations are authored by Aitchison while I cannot remember them exactly.*

We agree with the reviewer that the source on line 187 did not discuss the distributional properties of CBEA. We have amended the citation with the source from Aitchison and Shen [11] which provides details on the logistic normal distribution for compositional data and the original source from Egozcue [12] which talks about the relationship between ILR coordinates and the ALR coordinates that motivated the logistic normal distribution mentioned above.

J. *On line 536 the authors mention "inflated counts", I have no idea what this means.*

We have updated the simulation design section (now in supplementary analyses) to detail the meaning of “inflated counts”. We have also amended this confusing language and refer to sets that have a higher mean abundance as “enriched”. In essence, “inflated counts” refers to when sets (or individual taxon) have a fold change increase in absolute counts in a certain condition (e.g. IBD) compared to control. This is equivalent terminology to refer to taxa that are differentially abundant across conditions, but also refers specifically to the mechanism of abundance difference (fold change).

K. *Lines 572 to 582. I don't understand how this hypothesis makes sense. How does taxa-specific bias relate to the performance of DESeq2 or corncob? The writing here is poor. Also, I am not sure how this could be, are you not basing the gold-standard truth off of permuted data which you know has no signal? This permutation would maintain the measurement bias ... as a result it would seem the data does not support this hypothesis. I expect I am missing something.*

We would like to thank the reviewer for bringing this to our attention. We have tweaked the manuscript (see discussion section) to hopefully add further clarity to this issue. Here, we provide the discussion that is included in our revision.

The comments referenced by the reviewer were referring to the difference in performance of corncob and DESeq2 between simulations and real data analysis. In the simulation analysis, these methods show low type I error and low power, while conversely in real data analyses (i.e., the permutation analyses) these methods show high type I error and high power (when compared against CBEA). We did not expect this result, which indicates that there are systematic differences between our assumption of the data generating process in the design of the simulations and data from real world sources. In this section we specifically hypothesize the mechanism behind the differences between simulated and real data sets and deduced that it might be due to taxa-specific biases. According to McLaren et al. [13], sum-based aggregations are particularly sensitive to this type of bias. We agree with the reviewer that in permutation analyses using real data this bias would be preserved, which explains that high type I error observed when applying DESeq2 and corncob. However, this is something we did not consider in our simulation, hence the differences in performance.

2. *Modeling Choices*

A. *As far as I can tell the authors do not state how they are handling zeros. This is a non-trivial methodologic detail especially if they are simply taking log-ratio transforms of count data. To what extent is the non-normality of the authors results simply a product of directly transforming count data without accounting for the variance of the counts. For example, count data typically have a mean variance relationship that seems largely ignored by the authors approach. Moreover, there has been a number of advances in compositional modeling of microbiome focusing on Multinomial logistic normal models that are not addressed by the authors. In fact in light of the availability of these methods the authors modeling of these counts seems sub-par.*

We thank the reviewer for the comments. Our strategy for addressing sparsity in microbiome data is to use pseudocounts to ensure the validity of the log-ratio transformations. As such, we have stated this assumption more clearly in the methods formulation section of the revised manuscript.

We also acknowledge in our discussion section the limitations of this approach and mention alternative methods that users can apply prior to running CBEA. However, according to our experimental results, the performance of our approach is not particularly sensitive to the level of sparsity in the data.

As clarified above, we do not model the count data directly but rather model the relative proportions that result from a total sum normalization of the counts. Our approach is to perform an ILR-like transformation to the proportions corresponding to the set annotations and perform inference through empirical modeling of the test statistic under the null. Our simulation studies have demonstrated that the empirical distribution of our test statistic is well approximated by a normal distribution. Furthermore, real data analyses also show that a normal approximation generates good performance values for all considered situations.

As the reviewer noted, multinomial logistic normal models are useful for modeling count data direction, and it may be feasible to apply the multinomial logistic normal model to perform set-based enrichment analysis. Although we are not away of any existing approaches that utilizes this distribution for set-based testing, this is an interesting idea that we hope to explore in future research. We have added this as part of our "Limitations and future directions" section.

3. *Unsubstantiated Claims*
   There are a number of unsubstantiated claims where the language needs to be altered to be more precise.

A. *Line 493: "These results demonstrate that cILR generated scores are informative features in disease prediction tasks." No. These results demonstrate that cILR COULD be informative features in disease predictions tasks. I am not convinced that these are even useful for the case-studies shown in this manuscript let alone other tasks. Moreover, the comparison methods ssGSEA and GSVA seem like odd choices. Are the authors only using methods that can take set-based features? This does not account for the potential that the chosen sets are not informative. The later seems like an important case to establish the motivation of the current work.*
   We agree with the reviewer that the predictive capacity of set-based features would be low if the chosen sets are non-informative or not interesting. We have amended the statement above in the manuscript as suggested by the reviewer. We would like to clarify that in this manuscript we are agnostic as to how the sets are constructed and whether there is a performance increase using sets compared to using the basic features. What we demonstrate in the manuscript is the relative performance of the different approaches to aggregation in instances where the researcher decides aggregation is of interest. As such, our claim that "CBEA generated scores are informative features" refers to the fact that given the same sets of microbes, scores constructed by CBEA can be informative towards prediction compared to similar approaches, suggesting that it is valid to use set-based features generated using CBEA for prediction purposes. However, as stated above, we agree with the reviewer that this is a strong statement and have adjusted it accordingly. We also added the context provided in this response to the results interpretation in the manuscript (refer to "Downstream analysis using prediction models"/ "Disease prediction" sections).

In terms of the comparison methods of GSVA and ssGSEA, we were motivated in this section by the fact that CBEA generates scores at the sample level, performing as a transformation of a n x p matrix of *p* taxa and *n* samples into a *n x m* matrix of *m* sets and *n* samples. ssGSEA and GSVA are approaches which also calculates enrichment scores per sample using the original matrix and set annotation as inputs. As such, we felt it was appropriate to use these models as comparison points. The goal is that for predictive analyses, we can fit a model (in our case, a simple random forest) to these scores to perform predictive analysis using set-based features. In other words, ssGSEA, GSVA, and CBEA are all "feature engineering" approaches rather than outcome prediction models.

B. *Line 535-537. The authors show that their model displays Type 1 error control on a set of of simulated datasets. They make some claims about false-discovery control on real data on lines 397-406 but I really don't follow how they know what is non-random or random on this dataset. It seems like they have a strong hypothesis about aerobic vs. anaerobic but that hypothesis seems too weak to serve as a gold-standard reference. Overall these claims are unsubstantiated. I would emphasize that any claim saying a model can be trusted is suspect and bordering on overtly false -- any model can fail and nearly all models are mis-specified there are times at which a model may be useful but that is about it. No model can be globally trusted.*
   We thank the reviewer for raising this issue. For the simulation results (now in the supplementary materials), we updated the manuscript to specify the precise design of our simulation experiments and to clarify that these scenarios represent only a subset of the data sets that users may encounter in practice. As such, we have modified our performance claims in the supplementary analyses where this is relevant.

   In terms of our real data evaluation, we have adjusted our type I error analyses for sample level inference to using randomly generated sets instead (as suggested by the source referred by reviewer 2). We believe this better represents the null distribution stated in CBEA and addresses the reviewer's concern over what is random and non-random in the data set. For power analyses, we agree that the aerobic vs anaerobic hypothesis is not strong enough to serve as ground truth. As a result, we have clarified and provided further discussion on this aspect of the evaluation, and provide discussion on the lack of standardized data sets for enrichment testing evaluation. Despite these limitations, our analyses based on the aerobic vs. anaerobic label can still provide good insight into model performance since the hypothesis does have a clear and straightforward biological interpretation (i.e. based on easy to determine natural characteristics of the microbes) and has been used in prior manuscripts that attempt to validate differential abundance analyses for microbiome data [14].

4. *Other Comments on Clarity*

A. *The writing after line 215 lacks detail. I kept waiting for a methods section to answer some of my questions (e.g., how was mu or phi chosen in equation 3) but these don't seem to be listed anywhere. Details in the remaining parts of the manuscript are inconsistently given or vague. e.g., Line 249 "all sample sizes were set to 10,000". Do you mean sequencing depth? Number of reads? Number of technical replicates? What is this referring to?*
   We have adjusted the "Evaluation" section of the manuscript to improve readability and fill in missing gaps in our evaluation methodology. The simulation section in the revised supplementary analyses also have clearer descriptions of the relevant simulation design. With regards to the specific examples provided by the reviewer, we provide some clarification as follows:

   1) The μ and ϕ parameters were chosen by fitting a negative binomial distribution (using maximum likelihood approach with the *fitdistrplus* package [15]) on non-zero entries in each taxon in the human microbiome 16S data set. The median values across all estimates were chosen as the final estimates for the simulation procedure.

   2) 10,000 samples refers to the number of samples (i.e. the number of biological replicates). Since we're attempting to perform inference per sample (assign a p-value per sample), this is equivalent to the number of hypotheses tested for our enrichment analysis procedure.

B. *The writing after line 215 is hard to read. In part this relates to the lack of detail but I think it also stems from the fact that the manuscript starts being written in triplicate for Single Sample Enrichment, Differential Abundance Analysis, and then Prediction. It makes the paper repetitive and hard to follow. Further, figures are repetitive and poorly labeled so it's hard, at a glance, to figure out what figure links to what part of the paper. I have never seen a discussion written in*

the parts but this just adds to the feeling that this is just a paper written in triplicate without a coherent message beyond the initial idea which ends around line 215. Further it was not clear from reading the introduction that the paper would be organized like this; some warning in the introduction may help a bit. In fact, it was not even clear what the distinction between single-sample enrichment and differential abundance was from the introduction. In addition, this notation is non-standard. Typically enrichment (e.g., as used in gsEa) refers to essentially differential expression but for sets of genes (i.e., it is typically a comparison between groups). This makes the "single-sample enrichment" terminology confusing.
To address structural confusion, we have included an overview of the manuscript in the introduction. We also restructured the manuscript into different evaluation criteria instead of analysis tasks (refer to the preamble of this response letter). We also adjusted figure labels and captions for clarity across different sections.

We agree with the reviewer that enrichment testing is essentially differential abundance for set-based features. As such, we have combined "single-sample" and "differential abundance" into the same type of analysis (but now broken down by evaluation type such as type I error evaluation and power). "Single sample" is now referred to as "sample-level inference" where we are testing for enriched sets within the sample (significantly enriched compared to background taxa only for that sample), which is an unsupervised (i.e. without using sample labels) test similar to VAM [16]. "Differential abundance" is now referred to as "population-level inference" where we test for differences in enrichment scores across case/control status, which is a supervised approach similar to GSEA [17].

Reviewer 2:

<u>*Summary*</u>

Nguyen et al. present a new method for taxonomic enrichment analysis of microbiome data based on an isometric log-ratio transformation of compositional and the competitive null hypothesis borrowed from the gene set enrichment literature. The main strengths are a well-written and structured manuscript, a solid statistical and analytical foundation of the method, and a thorough evaluation of the method on simulated and real datasets. The main weaknesses are installation issues with the R companion package, a lack of adaptation of existing standards for the benchmarking of gene set enrichment methods, and a number of theoretical considerations with the use of a competitive null hypothesis for enrichment testing.

We would like to thank Reviewer 2 for the detailed and encouraging responses. We have made structural changes in the manuscript (as described in the preamble section of this response) to make sure we acknowledge the standards set out by Geistlinger et al. 2021 [3]. Additionally, we have added a discussion on adjusting for correlation, and have also fixed the R package.

1. *Installation:*

Using a recent R installation (R.4.1.0) and Bioconductor installation (3.13), I was not able to install the package. The error message and my session info is included below. The method looks useful for the community and I would strongly encourage a Bioconductor submission (or at least a CRAN submission) of the package to ensure that the package passes R CMD build, check, and install in a continuous integration setup. (Detailed error message that was attached by Reviewer 2 was excluded from this response for clarity)

We have provided an updated version of the package and submitted it to CRAN/Bioconductor (https://github.com/Bioconductor/Contributions/issues/2449). The current in development version on GitHub has passed R CMD CHECK --as-cran on Windows, MacOS, and Linux (Ubuntu 20.08) via GitHub Actions. If there are any installation issues, please let us know.

2. Adapting standards for the benchmarking of enrichment methods:

Geistlinger et al. (doi: 10.1093/bib/bbz158) has recently introduced an extensible framework for reproducible benchmarking of enrichment methods based on defined criteria for applicability, gene set prioritization and detection of relevant processes. This setup consists of compendia of curated and standardized datasets and a number of criteria that apply as-is also for new enrichment methods in the microbiome data realm (such as runtime, proportion of rejected null hypotheses, behavior on permuted sample labels and random gene sets). Although I would really like to commend the authors for using curated and standardized datasets from curatedMetagenomicData and HMP16SData, the authors then proceed with the practice of self-assessment over various scenarios which is typically difficult to transport and apply for new methods. Being one of the first methods for enrichment analysis in the microbiome realm (but very likely not the last one), the paper has the opportunity to very early on set the baseline for how new enrichment methods in the microbiome space should be evaluated building on lessons learned in the gene set enrichment literature. This could be achieved (a) clearly communicating the existence of such standards, (b) adapting existing standards where possible, and (c) to point out where adaption of such standards would require further work, as there might well be criteria that do not straightforward translate from gene set enrichment to taxon set enrichment.

We thank the reviewer for directing us to the paper by Geistlinger et al. [3]. We agree that set-based approaches from the microbiome field should learn and adapt from existing standards from the gene-set testing literature. After consulting Geistlinger et al, we noticed that many of the existing sections of the manuscript already correspond to the structure recommended in the Geistliner et al. paper. As such, we have reorganized the manuscript to properly communicate the relationship between our evaluation strategy and the standards set by Geistlinger et al. These changes are summarized in the pre-amble section of the reviewer response.

We hope this reorganization will enable more direct comparisons between existing standards in the gene set testing literature and our own evaluation strategy.

3. On the use of the competitive null hypothesis for enrichment testing:

The authors demonstrate that cILR controls for type I error even under high sparsity and high inter-taxa correlation. However, it has been pointed out that strict type I error rate control might not be a desirable feature for enrichment methods (Goeman and Buhlman, 2009; Wu and Smyth, 2012; Geistlinger et al. 2021). Gene set enrichment analysis is an exploratory process, not a confirmatory, diagnostic process, where strict type I error control augments the lack in power which is well documented for competitive enrichment testing (Goeman and Buhlman, 2009; Wu and Smyth, 2012; Geistlinger et al. 2021) and as the authors demonstrate in their own evaluations. Furthermore, Geistlinger et al. 2021 (Figure 4 therein) has demonstrated that despite controlling the type I error rate, methods might demonstrate widely different rejection rates on real datasets. It is in this context noteworthy that the authors of Camera (Wu and Smyth, 2012), which deliberately abandons strict type I error control by default to compensate for the apparent lack in power of competitive methods.

We agree with the reviewer on the overall goal of enrichment analysis and the discussions presented in the existing literature on the trade-off between power and type I error control. We agree that detecting highly correlated taxa sets can have biological importance (as discussed in Wu and Smyth [18]) and have clarified that the decision for strict type I error control is up to the user. We have also provided additional discussion in the “Statistical properties” section about this topic, including a further clarification on the motivation behind adjusting for inter-taxa correlation.

Bibliography

1. Silverman JD, Washburne AD, Mukherjee S, David LA. A phylogenetic transform enhances analysis of compositional microbiota data. Fodor A, editor. eLife. 2017;6: e21887. doi:10.7554/eLife.21887

2. Washburne AD, Silverman JD, Leff JW, Bennett DJ, Darcy JL. Phylogenetic factorization of compositional data yields lineage-level associations in microbiome datasets. PeerJ. 2017; 26.

3. Geistlinger L, Csaba G, Santarelli M, Ramos M, Schiffer L, Turaga N, et al. Toward a gold standard for benchmarking gene set enrichment analysis. Briefings in Bioinformatics. 2021;22: 545–556. doi:10.1093/bib/bbz158

4. Aitchison J. The Statistical Analysis of Compositional Data. Journal of the Royal Statistical Society: Series B (Methodological). 1982;44: 139–160. doi:10.1111/j.2517-6161.1982.tb01195.x

5. Gloor GB, Macklaim JM, Pawlowsky-Glahn V, Egozcue JJ. Microbiome Datasets Are Compositional: And This Is Not Optional. Front Microbiol. 2017;8. doi:10.3389/fmicb.2017.02224

6. Quinn TP, Erb I, Richardson MF, Crowley TM. Understanding sequencing data as compositions: an outlook and review. Bioinformatics. 2018;34: 2870–2878. doi:10.1093/bioinformatics/bty175

7. Weiss S, Xu ZZ, Peddada S, Amir A, Bittinger K, Gonzalez A, et al. Normalization and microbial differential abundance strategies depend upon data characteristics. Microbiome. 2017;5. doi:10.1186/s40168-017-0237-y

8. McMurdie PJ, Holmes S. Waste Not, Want Not: Why Rarefying Microbiome Data Is Inadmissible. PLOS Computational Biology. 2014;10: e1003531. doi:10.1371/journal.pcbi.1003531

9. McKnight DT, Huerlimann R, Bower DS, Schwarzkopf L, Alford RA, Zenger KR. Methods for normalizing microbiome data: An ecological perspective. Methods in Ecology and Evolution. 2019;10: 389–400. doi:10.1111/2041-210X.13115

10. Egozcue JJ, Pawlowsky-Glahn V. Groups of Parts and Their Balances in Compositional Data Analysis. Mathematical Geology. 2005;37: 795–828. doi:10.1007/s11004-005-7381-9

11. Aitchison J, Shen SM. Logistic-normal distributions:Some properties and uses. Biometrika. 1980;67: 261–272. doi:10.1093/biomet/67.2.261

12. Egozcue JJ, Pawlowsky-Glahn V, Mateu-Figueras G, Barceló-Vidal C. Isometric Logratio Transformations for Compositional Data Analysis. Mathematical Geology. 2003;35: 279–300. doi:10.1023/A:1023818214614

13. McLaren MR, Willis AD, Callahan BJ. Consistent and correctable bias in metagenomic sequencing experiments. Turnbaugh P, Garrett WS, Turnbaugh P, Quince C, Gibbons S, editors. eLife. 2019;8: e46923. doi:10.7554/eLife.46923

14. Calgaro M, Romualdi C, Waldron L, Risso D, Vitulo N. Assessment of statistical methods from single cell, bulk RNA-seq, and metagenomics applied to microbiome data. Genome Biology. 2020;21: 191. doi:10.1186/s13059-020-02104-1

15. Delignette-Muller ML, Dutang C. fitdistrplus: An R package for fitting distributions. Journal of Statistical Software. 2015;64: 1–34.

16. Frost HR. Variance-adjusted Mahalanobis (VAM): a fast and accurate method for cell-specific gene set scoring. Nucleic Acids Research. 2020;48: e94–e94. doi:10.1093/nar/gkaa582

17. Subramanian A, Tamayo P, Mootha VK, Mukherjee S, Ebert BL, Gillette MA, et al. Gene set enrichment analysis: A knowledge-based approach for interpreting genome-wide expression profiles. PNAS. 2005;102: 15545–15550. doi:10.1073/pnas.0506580102

18. Wu D, Smyth GK. Camera: a competitive gene set test accounting for inter-gene correlation. Nucleic Acids Res. 2012;40: e133. doi:10.1093/nar/gks461
