# Supplementary material for: CBEA: Competitive balances for taxonomic enrichment analysis
Source: PLoS Comput Biol. 2022 May 18;18(5):e1010091. doi: 10.1371/journal.pcbi.1010091 (PMC9154102; doi:10.1371/journal.pcbi.1010091)
Supplement: Attachment — Submitted filename: CBEA_CoverLetter.pdf [file pcbi.1010091.s010.pdf]

Dartmouth
GEISEL SCHOOL OF MEDICINE

March 28, 2022

PLOS Computational Biology

Dear PLOS Computational Biology Editors:

Please find enclosed our revised manuscript entitled "CBEA: Competitive balances for taxonomic enrichment analysis” (PCOMPBIOL-D-21-01648R1).

This revision addresses the single outstanding comment from Reviewer 2:

My comments regarding the manuscript text have been satisfactorily addressed. The authors also indicate that they started a Bioconductor submission under https://github.com/Bioconductor/Contributions/issues/2449. However, the submission under the provided link has been closed due to inactivity / revisions performed to the manuscript and implementation. An initial review by a reviewer from the Bioc core team indicates that the package is in good shape and would only need minor modifications for acceptance in Bioconductor. As I assume this new method to be a useful contribution for the community, I would thus strongly encourage to follow through with the Bioconductor submission and include a link to the Bioconductor package in the manuscript (once the package has passed review and is accepted in Bioconductor).

The CBEA package has now been accepted by Bioconductor (see https://github.com/Bioconductor/Contributions/issues/2449) and will be officially released on 4/30. The revised manuscript has been updated to refer to the CBEA Bioconductor package rather than the GitHub project.

We confirm that this work is original and, aside from the bioRxiv preprint, has not been submitted or published elsewhere. All authors have reviewed and approved the submission of this manuscript. We do not have any competing interests to declare.

Sincerely,

H. Robert Frost, Ph.D.
Assistant Professor of Biomedical Data Science
Geisel School of Medicine at Dartmouth
Phone: 603-667-1884
Email: rob.frost@dartmouth.edu
